# Les Larves de Culicidae et Leur Dynamique Saisonnière dans la Région de Fès-Meknès, Maroc

**DOI:** 10.48327/mtsibulletin.n1.2021.89

**Published:** 2021-04-12

**Authors:** T. Filali Mouatassem, A. El Ouali Lalami, C. Faraj, N. Rais, R. Guemmouh

**Affiliations:** 1 Laboratoire de biotechnologie, conservation et développement des ressources naturelles, Faculté des sciences Dhar El Mahraz, Fès, Maroc; 2 Institut supérieur des professions infirmières et techniques de santé Fès, Direction régionale de la Santé, Hôpital EL Ghassani, Fès, Maroc; 3 Laboratoire d'entomologie médicale, Institut national d'hygiène, Rabat, Maroc; 4 Laboratoire de physique appliquée, informatique et statistique, Faculté des sciences Dhar El Mahraz, Fès, Maroc

**Keywords:** Faune culicidienne, Fluctuation saisonnière, *Culex pipiens*, Cx. perexiguus, Cx. theileri, Cx. hortensis, Culiseta longiareolata, Uranotaenia unguiculata, Anopheles cinereus, *An. maculipennis* s. l., *An. sergentii*, Jnan EL Alami, Barrage Lgaâda, Awinat Elhajaj, Source Douwar Lhandiya, Fez, Maroc, Maghreb, Afrique du Nord, Culicidian fauna, Seasonal fluctuation, *Culex pipiens*, Cx. perexiguus, Cx. theileri, Cx. hortensis, Culiseta longiareolata, Uranotaenia unguiculata, Anopheles cinereus, *An. maculipennis* s. l., *An. sergentii*, Jnan EL Alami, Lgaâda dam, Awinat Elhajaj, Douwar Lhandiya Source, Fez, Morocco, Maghreb, Northern Africa

## Abstract

**Objectif:**

Dans le cadre de la prévention des maladies vectorielles au centre du Maroc, une étude de la fluctuation saisonnière de la biodiversité culicidienne a été menée de novembre 2015 à novembre 2016 sur quatre sites de reproduction situés dans la ville de Fès au centre du Maroc (Jnan EL Alami, barrage de Lgaâda, Awinat Elhajaj, Source Douwar Lhandiya). L'étude visait à améliorer les connaissances sur la dynamique saisonnière et la période d'activité des moustiques culicidiens, afin de cibler la période de lutte.

**Méthodes:**

Les larves ont été collectées par la méthode du dipping à un intervalle de 15 jours, au moins une fois par mois. Les analyses statistiques ont été réalisées à l'aide de la version 3.6.1 du logiciel de traitement statistique R.

**Résultats:**

Au cours de cette étude, neuf espèces ont été trouvées avec de fortes variations mensuelles et saisonnières du nombre de chaque espèce et d'un site à l'autre. Les espèces prédominantes sont des vecteurs de maladies: *Culex pipiens, Cx. perexiguus, Cx. theileri*, vecteurs connus du virus du Nil occidental, suivis par *Anopheles maculipennis* s. l. et *An. sergentii* qui sont les principaux vecteurs du paludisme au Maroc. *Cx. pipiens* et *Cx. perexiguus* ont atteint la plus forte densité en septembre, tandis que *Cx. theileri* a été trouvé en grand nombre en février et peut donc émerger à la fin de l'hiver et au milieu du printemps. Le nombre le plus faible d'*An. sergentii* a été collecté en novembre, mais il a augmenté en septembre, octobre et décembre. *An. maculipennis* s. l. est apparu en juin, avec des récurrences en mars et juillet. *Culiseta longiareolata* a été trouvé au printemps et en été et en abondance en juin. Cependant, *Uranotaenia unguiculata* n'était présent qu'en septembre et octobre. *An. cinereus* et *Cx. hortensis* étaient tous les deux présents en novembre et février.

**Conclusion:**

Les résultats obtenus seront un outil important pour la gestion et le suivi de la prolifération des Culicidae et pourront être utilisés pour améliorer l'efficacité de la gestion de la lutte.

## Abstract (en)

**Objective:**

As part of the prevention of vector-borne diseases in central Morocco, a study of the seasonal fluctuation of Culicidian biodiversity was conducted from November 2015 to November 2016 on four breeding sites located in the city of Fez in central Morocco (Jnan El Alami, Lgaâda dam, Awinat Elhajaj, Douwar Lhandiya Source). The study aimed to improve the knowledge of seasonal dynamics and activity period of Culicidian mosquitoes, in order to target the period of control.

**Methods:**

Larvae were collected by dipping method at a 15-day interval at least once a month. Statistical analyses were performed using version 3.6.1 of the statistical processing software.

**Results:**

During this study, nine species were found with high monthly and seasonal variations in numbers of each species and from site to site. Predominant species are vectors of disease: *Culex pipiens, Cx. perexiguus, Cx. theileri*, known vectors of West Nile virus, followed by *Anopheles maculipennis* s. l. and *An. sergentii* that are the main vectors of malaria in Morocco. *Cx. pipiens* and *Cx. perexiguus* reached the highest density in September, while *Cx. theileri* was found in large numbers in February and can therefore emerge in late winter and mid-spring. The lowest number of *An. sergentii* was collected in November, but increased in September, October and December. *An. maculipennis* s. l. occurred in June, with recurrences in March and July. *Culiseta longiareolata* was found in spring and summer and in abundance in June. However, *Uranotaenia unguiculata* was only present in September and October. Both *An. cinereus* and *Cx. hortensis* were present in November and February.

**Conclusion:**

The results obtained will be an important tool for management and monitoring Culicidae proliferation and can be used to improve the efficiency of control management.

## Introduction

Au Maroc, *Culex pipiens* a été considéré comme vecteur du virus West Nile. *Anopheles labranchiae* a été identifié comme espèce responsable de la transmission du paludisme.

La surveillance et la lutte contre la prolifération de ces espèces vectrices d'agents infectieux ne peuvent être efficaces sans la bonne connaissance de leur évolution spatio-temporelle. C'est dans ce cadre que s'inscrit cette étude menée dans la région de Fès-Meknès, Centre Nord du Maroc.

L'obtention de nouvelles données sur la dynamique saisonnière des espèces, autres que celles de l'inventaire quantitatif [4] et que celles des paramètres biotiques et abiotiques affectant leurs proliférations, leurs abondances et leurs répartitions [3], sera utile pour élaborer une stratégie de suivi des moustiques dans cette zone. Cette étude originale, sera d'un grand intérêt pour les responsables du programme de lutte contre les maladies à transmission vectorielle aux niveaux régional et national.

## Matériel et Méthodes

### Milieu d'étude

La ville de Fès est située au centre nord du Maroc à 415 m d'altitude sur les terres fertiles de la plaine du Saïs, entre les riches forêts des chaînes montagneuses du moyen Atlas et le Rif. Elle possède un climat continental.

### Gîtes étudiés

**Fig. 1:**
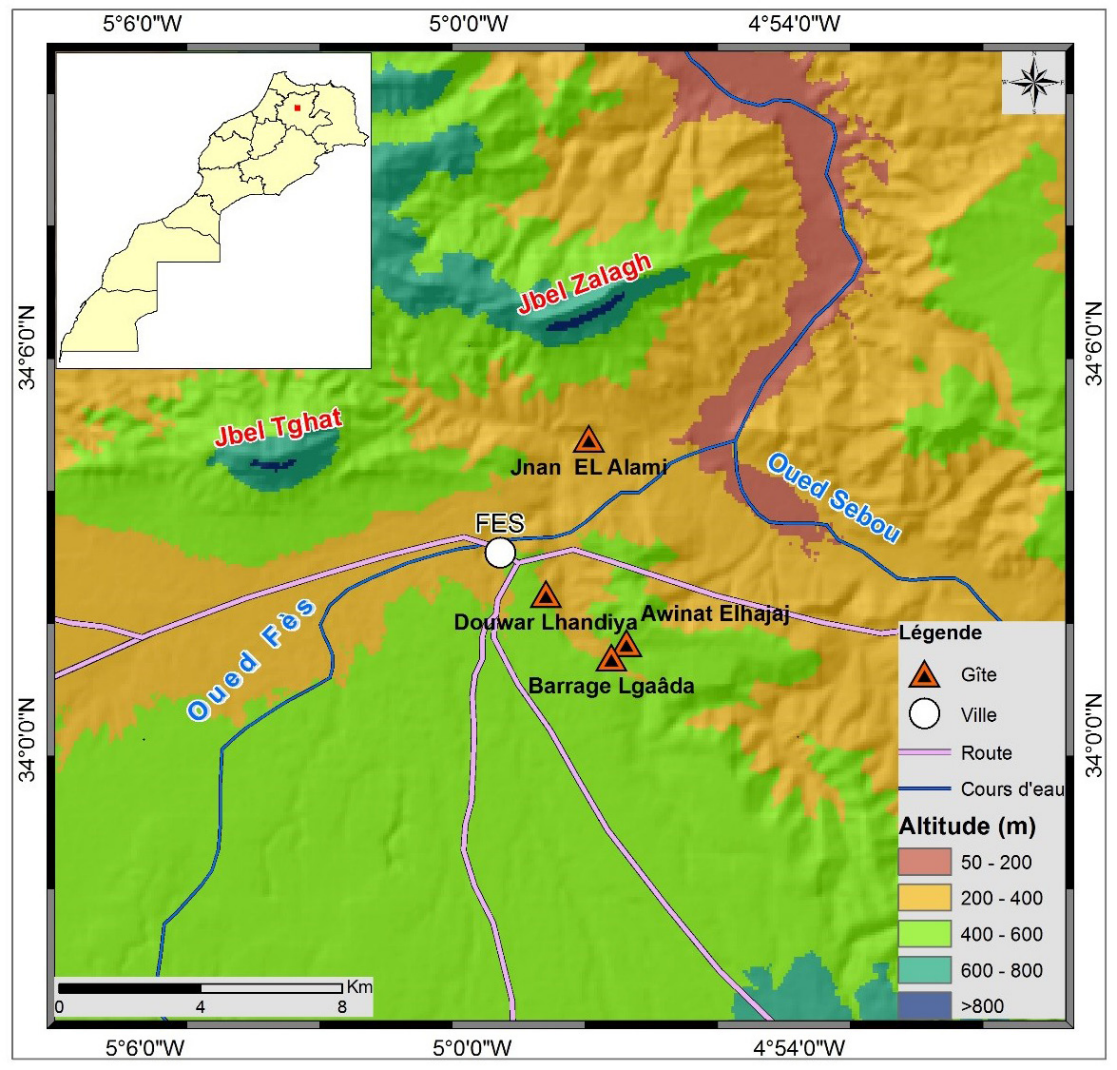
Localisation des quatre gîtes Location of the four collection sites

Le choix des habitats larvaires a été fait après une prospection sur le terrain. Quatre gîtes ont été sélectionnés: Barrage El Gaâda, rivière Jnan EL Alami, marécage Awinat Elhajaj et source Douwar Lhandiya (Fig. 1).

### Période de récolte et identification des larves

L'échantillonnage des larves de moustiques a été effectué, de novembre 2015 à novembre 2016, à un rythme quasi bimensuel, en effectuant 10 coups de louche d'une capacité de 250 millilitre. Les larves de premier, deuxième et troisième stades ont été ramenées au laboratoire à l'état frais et mis en élevage pour les identifier dès qu'elles ont mué en larves de quatrième stade. L'identification a été faite à l'aide de la clé d'identification marocaine et du logiciel d'identification de Bruhnes et al [1].

### Traitement des données

Les données ont été traitées avec la version 3.6.1 du logiciel de traitement statistique.

## Résultats

**Fig. 2:**
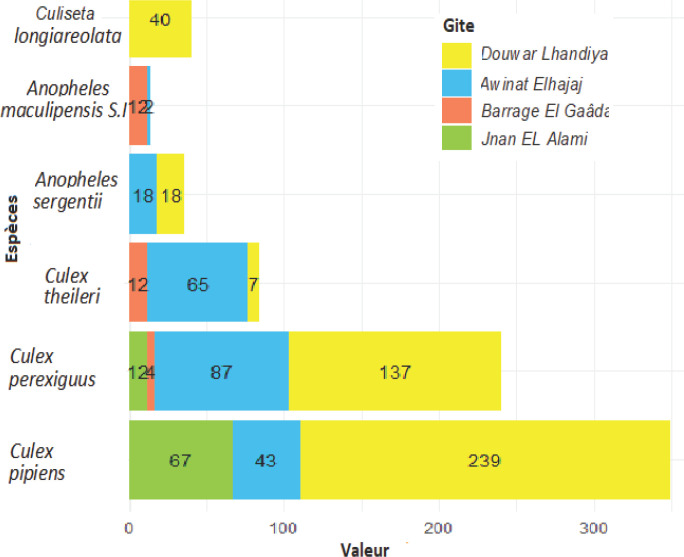
Barplot sous R horizontal des abondances relatives des espèces de Culicidae récoltées au niveau des gîtes représentatifs de la région de Fès (novembre 2015 à novembre 2016) Relative abundance of Culicidae species collected at representative breeding sites within the Fez region (November 2015 to November 2016)

Au total, 772 larves d'espèces culicidiennes ont été collectées. Les espèces les plus abondantes sont *Cx. pipiens* (Linnaeus, 1758) (45,1%) et *Cx. perexiguus* (Theobald, 1903) (31,2%). Les espèces les moins présentes sont *Cx. theileri* (Theobald, 1903) (10,9%) et *Cu. longiareolata* (Macquart, 1838) (5,2%) et les espèces rares sont *U. unguiculata* (Edwards, 1913) (0,8%), *An. cinereus* (0,3%) et *Cx. hortensis* (0,1%). Parmi les espèces récoltées, nous avons trouvé un vecteur connu du paludisme (*An. maculipennis* s. l. (1,8%) et un autre, *An. sergentii* (Theobald, 1907) (4,7%), suspecté d'intervenir dans sa transmission (Fig. 2). Les espèces rares *U. unguiculata, An. cinereus* et *Cx. hortensis* ne seront plus considérées par la suite.

La distribution spatio-temporelle des espèces n'est pas homogène (Tableau 1).

**Tableau I:** Matrice Mondrian des variations spatio-temporelles du nombre des Culicidés dans les gîtes d'étude Mondrian matrix of the spatial and temporal variations in the number of Culicidae in the study sites

**Figure:** 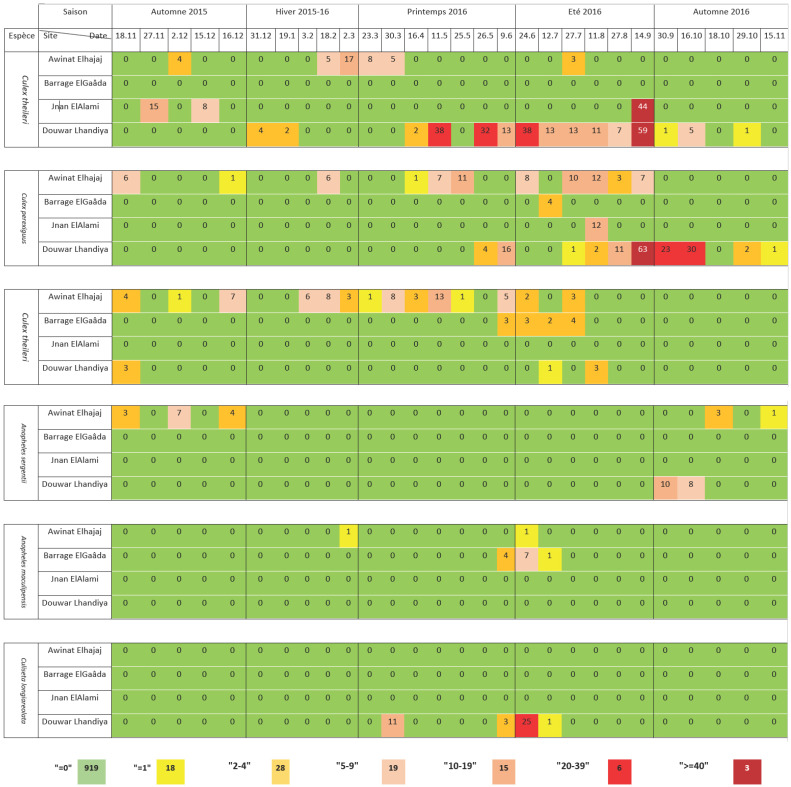

## Discussion

Les espèces trouvées font partie des espèces répertoriées au Maroc [5]. La grande majorité des larves a été observée au mois de septembre et la plus faible a été enregistrée en janvier.

Nos résultats ont montré également que *Cx. pipiens* présente une activité saisonnière continue. *An. maculipennis* s. l. a été présent de mars à juillet, qui est la période d'apparition des larves d'*An. labranchiae*, vecteur du paludisme autochtone qui coïncide à sa période de transmission au Maroc. Il s'avère que le mois de juillet est le plus propice pour cette espèce au niveau de la zone d'étude. *An. sergentii* a été présent en 2007 et 2008 durant les mois de la période sèche (juin, juillet et août) [2]. Nos observations ont aussi montré que cette espèce circule durant la saison humide (d'octobre jusqu'en décembre) et durant la saison sèche (septembre). L'évolution de la dynamique de ces deux espèces est peut-être due aux changements climatiques actuels. En effet, les changements climatiques pourraient entraîner des transmutations et des modifications dans la composition et la répartition spatio-temporelle des espèces ainsi que dans la dynamique de la propagation des maladies transmises par les moustiques. Ainsi, nous pouvons déduire que les espèces d'anophèles se sont adaptées aux différentes conditions climatiques.

Quoique que les collectes de larves ont été effectuées à partir d'un nombre limité de gîtes (4 gîtes) et que le nombre total de larves récoltées (772) est faible, ce qui peut être considéré comme une limite à cette étude, les résultats trouvés peuvent en partie étayer et aider les stratégies de santé publique à l'échelle locale et nationale.

## Conclusion

Nos résultats ont montré que les culicidés sont présents dans tous les sites d'études et à différents moments. Notre étude vient enrichir les données bibliographiques antérieures sur les périodes et les lieux d'apparition des vecteurs d'agents infectieux.

Des études complémentaires seraient nécessaires, pour confirmer nos résultats en collectant plus de spécimens au niveau d'autres écosystèmes et/ou avec d'autres techniques d'échantillonnage. Nous recommandons de poursuivre la nécessaire et primordiale surveillance entomologique spatio-temporelle dans le cadre de la prévention des maladies transmises par les vecteurs.

## Conflits D'intérêts

Les auteurs ne déclarent aucun conflit d'intérêts.
